# Mechanisms of Smoothened Regulation in Hedgehog Signaling

**DOI:** 10.3390/cells10082138

**Published:** 2021-08-20

**Authors:** Jie Zhang, Zulong Liu, Jianhang Jia

**Affiliations:** Markey Cancer Center, Department of Molecular and Cellular Biochemistry, University of Kentucky College of Medicine, Lexington, KY 40356, USA; jie.zhang3@uky.edu (J.Z.); zulong.liu@uky.edu (Z.L.)

**Keywords:** hedgehog signaling, smoothened, phosphorylation, ubiquitination, cholesterol, phospholipid

## Abstract

The seven-transmembrane protein, Smoothened (SMO), has shown to be critical for the hedgehog (HH) signal transduction on the cell membrane (and the cilium in vertebrates). SMO is subjected to multiple types of post-translational regulations, including phosphorylation, ubiquitination, and sumoylation, which alter SMO intracellular trafficking and cell surface accumulation. Recently, SMO is also shown to be regulated by small molecules, such as oxysterol, cholesterol, and phospholipid. The activity of SMO must be very well balanced by these different mechanisms in vivo because the malfunction of SMO will not only cause developmental defects in early stages, but also induce cancers in late stages. Here, we discuss the activation and inactivation of SMO by different mechanisms to better understand how SMO is regulated by the graded HH signaling activity that eventually governs distinct development outcomes.

## 1. Introduction

**Figure 1:**
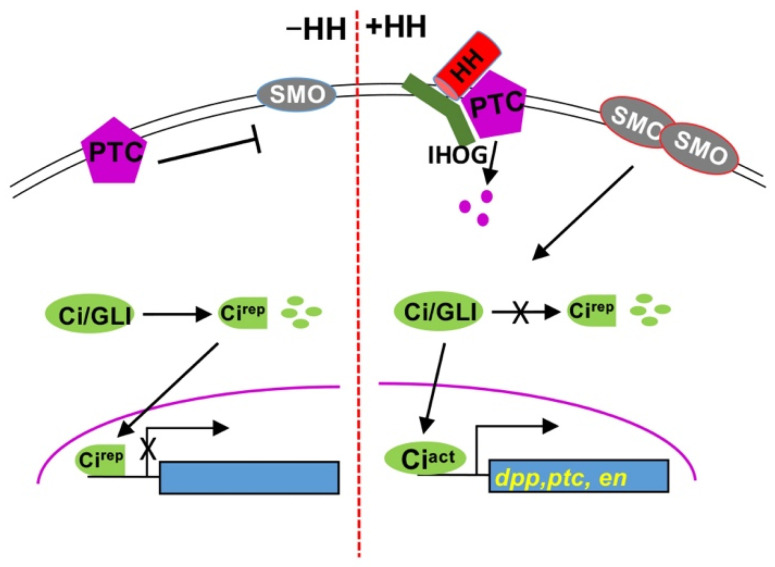
**The highly conserved HH signaling pathway.** Shown here is a scheme of the HH pathway. (**Left panel**), in the absence of HH, PTC inhibits SMO. Ci/GLI is processed into a truncated repressor form that enters the nucleus to block target gene expression. (**Right panel**), the presence of HH relieves the inhibition of PTC on SMO, leading to dimmerization and cell surface accumulation of SMO. Upon Hh stimulation, full-length Ci is activated to turn on target gene expression.

Hedgehog (HH) was initially discovered in *Drosophila* as a segment polarity gene involved in embryo patterning, and HH signaling pathways were shown to be very important in both embryonic development and post-developmental tissue homeostasis [1,2,3]. Aberrant HH signaling is implicated in many human disorders, including several types of cancers [4,5,6,7]. The HH signal is transduced by a signaling cascade that is highly conserved among different species. In *Drosophila*, at the plasma membrane, the HH receptor system includes the receptor complex patched (PTC)-interference HH (IHOG) and the signal transducer Smoothened (SMO) [8,9,10]. Binding of HH to PTC-IHOG relieves PTC inhibition on SMO, allowing SMO to block the partial degradation of the cubitus interruptus (Ci)/GLI family of zinc finger transcription factors and thereby induce the expression of HH target genes, such as *decapentaplegic* (*dpp*), *ptc*, and *engrailed* (*en*) [2,11] (Figure 1).

SMO, an atypical G protein-coupled receptor (GPCR), belongs to the family of Frizzled seven transmembrane proteins. SMO has three functional domains, an extracellular domain (ECD) containing a cysteine-rich domain (CRD), heptahelical transmembrane domains (TMDs), and a cytosolic tail at the C-terminus. The alignment of SMO sequences among species [12] indicated that the CRD and TMDs are highly conserved, with some diversity of amino acids in the C-terminal cytoplasmic tail (C-tail). It has also been shown that the CRD of SMO is critical for HH signaling in both *Drosophila* and vertebrates, and SMO lacking the CRD failed to accumulate on the plasma membrane or to the primary cilium [13,14]. The C-tail of SMO is otherwise critically regulated by phosphorylation that promotes the dimerization of the C-tail, thus the activation of SMO (see detailed discussion in below). The Frizzled family members often bind to ligands for signal transduction; however, SMO does not seem to have a ligand. SMO is subjected to various post-translational modifications, including phosphorylation, ubiquitination, and sumoylation. Different animal models have been used to study the mechanisms of SMO regulation as the mechanisms of SMO regulation are highly conserved among species [1,2,3,15].

Over the past decades, many HH pathway components have been identified; however, it is still unclear how PTC inhibits SMO to block the activation of the HH pathway. It is unlikely that PTC inhibits SMO by direct association [16,17]. In addition, the inhibition occurs even when SMO is present in 50-fold molar excess of PTC, and substochiometric levels of PTC can repress SMO activation [17,18]. These findings suggest that the inhibition process is catalytic [17]. The involvement of small molecules, rather than a protein ligand, has been proposed: PTC may inhibit the production of positive regulators or promote the synthesis of inhibitory molecules [17]. The mechanisms of SMO regulation bring a lot interest in the SMO research field because abnormal SMO activation often results in basal cell carcinoma and medulloblastoma, and SMO has been an attractive therapeutic target [19,20], exemplified by the U.S. FDA-approved drugs, such as vismodegib, sonidegib, and glasdegib, for the treatment of cancers known to be driven by SMO activation [21,22,23,24]. Interestingly, SMO may acquire drug resistance through a single amino acid mutation [25,26]. Taken together, a better understanding of the mechanisms that drive SMO activation/inactivation will not only provide insights into fundamental developmental processes, but also lead to new diagnostic tools and therapeutic approaches. This review will mainly focus on the regulation of *Drosophila* SMO; however, we also briefly discuss the differences between *Drosophila* and vertebrates.

## 2. SMO Phosphorylation, Dimerization, and Conformational Change

SMO is subjected to G protein [27] and phosphorylation regulations that control the switch between on and off and, therefore, the different signaling states of SMO. HH induces phosphorylation of SMO C-tail by multiple kinases, including protein kinase A (PKA) and casein kinase 1 (CK1) [28,29,30], casein kinase 2 (CK2) [31], G protein-coupled receptor kinase 2 (GPRK2) [32], and atypical PKC (aPKC) [33], which activate SMO by inducing differential phosphorylation [34], thus the gradual conformational change in the protein [34,35]. How numerous phosphorylation contribute to SMO activation? It is obvious that SMO adopts a differential phosphorylation mechanism in response to different levels of HH signaling activity [34]. In vertebrates, HH signal transduction depends on the primary cilium, and ciliary accumulation is required for SMO activation [36,37,38,39]. Similarly, phosphorylation by multiple kinases promotes the ciliary localization of mammalian SMO [40]. The phosphorylation of SMO has been reviewed [41,42]. Recent findings further suggest that HH promotes the association of the kinases with SMO [43], although the interaction can be dynamic. Which, out of SMO phosphorylation and cell surface accumulation, comes first? SMO cell surface accumulation turns out to be dynamic and progressive because HH makes changes in SMO subcellular localization in order for SMO to be associated with plasma membrane localized kinases [44], which is thought to account for the high-threshold HH signaling activity. Of note, phosphorylation mediates the balance between the transcriptional activator and repressor forms of Ci/GLI, which play dual roles by two distinct forms [41]. Fused (FU) and Costal 2 (COS2) are regulators of HH signaling downstream of SMO, and are also subjected to phosphorylation; however, we will not discuss this aspect because we are focusing on the mechanisms of SMO regulation.

Dephosphorylation processes are also involved in SMO regulation. Early studies identified protein phosphatase 2A (PP2A) as a positive regulator in HH signaling [45,46,47]. Later studies showed that protein phosphatase 4 (PP4) and PP2A were the phosphatases involved in regulating dephosphorylation of SMO and Ci, respectively [48]. The other independent study found that PP2A may also play a role in dephosphorylating SMO [49]. The phenotypes for the positive role of PP2A in HH signaling may mainly come from its activity in regulating Ci. It is not surprising that SMO can be regulated by different phosphotases that counteracts the kinases to phosohorylate SMO. A recent study showed that the protein phosphatase V (PPV) destabilizes widerborst (WDB), a regulatory subunit of PP2A, through competitive interaction with the catalytic subunit of PP2A, resulting in Wdb ubiquitination and degradation [50]. It is not very clear whether SMO can be regulated by specific phosphatases under different circumstances.

## 3. SMO Ubiquitination, Sumoylation, and Stability Control

**Figure 2:**
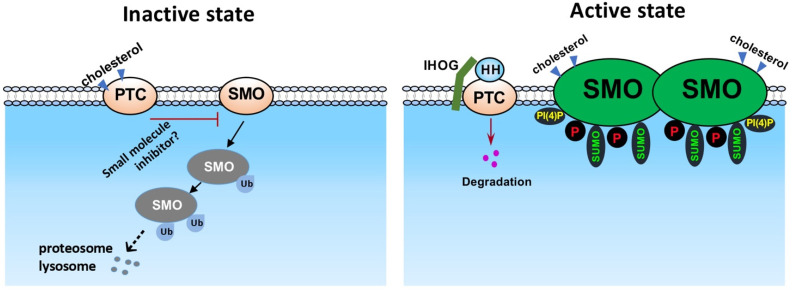
**SMO regulation in HH signaling.** Shown here is a scheme of SMO regulation through different mechanisms. In the absence of HH, unphosphorylated SMO is ubiquitinated and degraded through proteosomes and lysosomes. In the presence of HH, SMO is phosphorylated by multiple kinases. Lysine residues in the C-tail of SMO are sumoylated to counteract ubiquitination. Phosphorylation and dimmerization leads to the cell surface accumulation and activation of SMO. Both PI(4)P and chelesterol can activate SMO upon HH stimulation.

What is the mechanism to counteract SMO activation by phosphorylation? Studies using the *Drosophila* model have shown that ubiquitination promotes SMO degradation through lysosome- and proteasome-mediated pathways [51,52] (Figure 2). The initial experiments found that the inactivation of the ubiquitin-activating enzyme, UBA1, robustly accumulate SMO in *Drosophila* wing, indicating the blockade of SMO degradation [51,52]. More efforts than expected have been implemented to identify the E3 ubiquitin-protein ligase that specifically promotes SMO ubiquitination, likely because of the fact that SMO has many lysine residues that can be ubiquitinated, especially those lysine residues in the C-tail, or that multiple E3 ligases are involved. Studies have found that Smurf family E3 ubiquitin ligases target both SMO and PTC, and that HH promotes PTC ubiquitylation by releasing the Smurf family of E3s from SMO [53]. CUL4-DDB1 E3 ubiquitin ligase complex can also interact with and promote the ubiquitination in SMO C-tail [54]. Furthermore, Herc4 has also been identified as a E3 ligase of SMO [55] (our unpublished observations). To put SMO ubiquitination in the context of HH regulation, it has been shown that HH signaling promotes the phosphorylation of DDB1 by PKA and the dissociation of CUL4-DDB1 complex from SMO, resulting in the accumulation of SMO protein [54]. The other major regulation of SMO ubiquitination is the deubiquitination process. It has been well characterized that ubiquitin-specific protease 8 (USP8) promotes the removal of ubiquitin from SMO, and that HH stimulation promotes the accessibility of the deubiquitinase to SMO [51,52]. UCHL5 may also be involved in the deubiquitination process [56]. In these studies, the deubiquitination of SMO promotes the cell surface accumulation and thus the activation of SMO, suggesting that the removal of ubiquitin stabilizes SMO protein and therefore activates SMO on the cell surface.

In vertebrate systems, the transmembrane proteins, i.e., multiple epidermal growth factor-like domain 8 (MEGF8) and the RING family E3 ligase mahogunin ring finger 1 (MGRN1), form a receptor-like ubiquitin ligase complex on the cell membrane to promote SMO ubiquitination, endocytosis, and subsequent degradation [57]. Mice with MEGF8 or MGRN1 mutation shows specific defects in heart, limb, and skeleton. The treatment of the SMO inhibitor, vismodegib, can partially rescue the defects, suggesting that the phenotypes are caused by SMO activation. It is possible that the vertebrates use additional E3 ligase(s) to prevent SMO activation, and/or the vertebrates use different mechanisms in different developmental tissues. It is possible that different E3 ligases are involved in different aspects of SMO regulation in a vertebrate system, exemplified by a recent study in which the E3 ubiquitin ligase WWP1 specifically promotes the ubiquitination and the ciliary dynamics of vertebrate SMO [58]. Further studies are expected to identify and characterize the E3 ligases in vertebrate systems, similar to those E3 ligases involved in *Drosophila* SMO regulation.

SMO is also subjected to other post-translational modifications, such as sumoylation (Figure 2). Recent studies have found that the small ubiquitin-related modifier (SUMO) pathway components UBC9 (a SUMO-conjugating enzyme E2), PIAS (a SUMO-protein ligase E3), and Smt3 (the SUMO isoform in *Drosophila*) exhibit positive effects on SMO accumulation [59,60]. HH induces the sumoylation of SMO by dissociating the desumoylation enzyme ULP1 from SMO. Sumoylation, in parallel to phosphorylation, stabilizes SMO and activates HH signaling. Furthermore, KRZ, the *Drosophila* β-arrestin 2, blocks SMO sumoylation and prevents SMO accumulation. RNAi of KRZ decreases the interaction between SMO and ULP1 [60], suggesting that KRZ regulates the sumoylation of SMO through facilitating SMO-ULP1 interaction.

Phosphorylation by multiple kinases increases the level of SMO, whereas ubiquitination by different E3 ligases decreases the level of SMO. In addition, sumoylation stabilizes SMO protein. All these mechanisms contribute to the stability control of SMO in response to HH stimulation. Ubiquitination clearly shares the lysine residues that are sumoylated, indicating that sumoylation counteracts ubiquitination to activate SMO. PKA and CK1 inhibit SMO ubiquitination, whereas the PKA inhibitor, H-89, or the CK1 inhibitor, CK1-7, induce SMO ubiquitination. In addition, phospho-mimetic mutation of SMO exhibits a remarkably low level of ubiquitination and a high level of stability [52]. These suggest that phosphorylation counteracts ubiquitination of SMO and indicate that the levels of SMO protein are sophisticatedly controlled in vivo so as to mediate different thresholds of HH signaling activity.

There seems to be more mechanisms involved in SMO stability control. For example, it has been shown that the hepatocyte growth factor-regulated tyrosine kinase substrate (HRS) interacts with SMO and promotes SMO ubiquitination [61]. In addition, the endosomal sorting complex required for transport complex-III (ESCRT-III) core subunits, VPS32 (also known as Shrub in *Drosophila*, SNF7 in yeast, and CHMP4 in mammal) and VPS20 (CHMP6 in mammals), intracellularly regulate SMO stability [62]. Surprisingly, SMO can be activated in ESCRT-III, which does not rely on HH stimulation and is not inhibited by PTC, indicating that SMO activation can occur in an HH- and PTC-independent manner when SMO protein is highly accumulated in specific compartment in the cell. Mechanistically, a Krz-mediated pathway, operating in parallel to endocytosis, directs SMO to the ESCRT-III/multivesicular body (MVB), leading to the high accumulation and activation of SMO [62]. It was not the first time to show the ESCRT function in HH signaling. The ESCRT machinery has been shown to regulate this secretion and, thus, the long-range HH signaling activity [63], suggesting that the ultimate activation of HH signaling targets by blocking the ESCRT-III are likely caused by different layers of regulation. It should also be noted that Krz plays different roles in regulating SMO intracellular trafficking and stability control, although β-arrestin 2 may function differently in vertebrate system.

## 4. SMO Regulation by Lipid-Based Modulators

It has been a puzzle whether SMO is activated by a protein ligand, or a ligand-like molecule. Independent studies have found that HH induces the production of phosphatidyl-inositol 4-phosphate (PI(4)P) in both *Drosophila* wing disc and cultured cell [64,65] (Figure 2, right panel). Interestingly, PI(4)P directly interacts with SMO through the arginine motifs in the C-tail and stimulates phosphorylation and dimerization of SMO, which induces SMO cell surface/cilium accumulation [64]. The pleckstrin homology (PH) domain of GPRK2 facilitates PI(4)P to activate SMO. It is speculated that the PTC sterol sensing domain (SSD) attracts PI(4)P and, thus, inhibits SMO activation by PI(4)P [64]. In another study using molecular dynamics simulation, phospholipids are shown to bind SMO in the intracellular portion [66]. To support the notion that lipids play positive roles in activating SMO and thus HH signaling, a study has found that HH activates phospholipase A2 (PLA2) to promote the ciliary localization of SMO [67]. Together, these studies indicate phospholipid may function as a signaling molecular between PTC and SMO; however, the ligand-like property of these phospholipids may need further characterization, although these phospholipids bind to the receptor inside the cell and, therefore, potentially to the intracellular ligands.

Phospholipids may also play different roles in different tissue. Inositol polyphosphate 5-phosphatase E (INPP5E), functioning to remove the 5-phosphate from PI(4,5)P2, PI(3,4,5)P3, and PI(3,5)P2, affects ciliary phosphatidylinositol trafficking and positively regulates HH signaling [68,69]. Inactivation of INPP5E in primary cilia of neural stem cells leads to the accumulation of PI(4,5)P2, resulting in the ciliary membrane accumulation of Gpr161 [70], which plays a negative role in the HH signaling. A recent study further characterized the role of INPP5E in HH signaling and demonstrated that the negative role of INPP5E in HH signaling was through regulation of the active and repressor forms of GLI proteins [71]. It might be possible that INPP5E regulates GLI processing in the neural tube but regulates other aspects of the HH signal transduction in other tissues.

**Figure 3:**
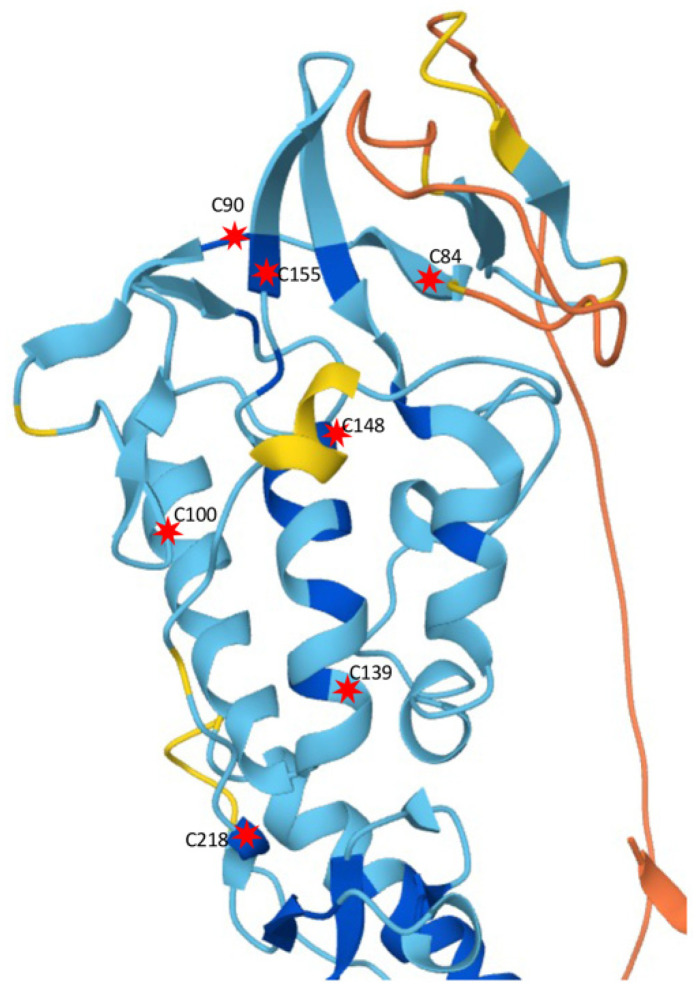
**SMO ECD structure.** Shown here is the structure of SMO ECD domain, adopted from the AlphaFold protein structure database. Critical cysteins are indicated by red stars with the positions numbered.

Extensive structural analyses have been performed to identify the possible extracellular ligand-like molecules that activate SMO. SMO contains a highly conserved extracellular CRD, but unlike other GPCRs, no ligand-binding function has been identified. It has been shown that SMO-mediated signal transduction is sensitive to sterols and oxysterol derivatives of cholesterol [72,73,74], and possibly sensitive to glucocorticoid budesonide [75]. In addition to the cysteins in the CRD, *Drosophila* Smo has other conserved cysteins (C218, C238, and C242) in ECD; mutating these cysteins significantly decreases the activity of SMO [75], suggesting that, in addition to the CRD, the cysteins in ECD play critical roles in maintaining the conformation and the activity of SMO. From our systematic analysis of recent data published [75,76] and data with different combinations of cysteine mutations (our unpublished data), it is likely that some of the cysteins in the ECD (including the CRD) play pivotal role(s) in SMO function (Figure 3). An interesting model has been proposed, in which specific molecules bind to the CRD, or even to the ECD, to induce a conformational change in the ECD, therefore, bringing the ECD to the TMDs and eventually change to the conformation of the TMDs and regulation of SMO signaling activity [75]. Recent studies have also suggested a direct involvement of oxysterol in the activation of HH signaling [74,77,78,79]; however, it is still unclear whether oxysterols are the endogenous SMO activator because of the significantly low physiological level than EC50 for HH pathway activation [74]. Unlike vertebrate SMO, *Drosophila* SMO CRD does not interact with oxysterols [80], raising the possibility of another endogenous ligand-like molecule to be commonly involved.

Exciting findings come from the recent structural studies, which suggest that cholesterol directly interacts with SMO [81] and activates SMO through binding to the extracellular CRD [76,82,83]; therefore, increasing the accessibility of cholesterol at the membrane of the cilium results in SMO activation [84]. However, an independent study indicates that the CRD domain is not required for PTC-SMO communication, and that cholesterol within the membrane bilayer is sufficient to activate SMO [85]. Cholesterol might also become covalently attached to the CRD under specific circumstances [82]. These seemingly contradictory models suggest important new lines of study to elucidate how cholesterol regulates SMO under physiological conditions. The structural studies may again give suggestions on how cholesterol regulates SMO. Another recent study indicates that SMO forms a π-cation lock in transmembrane domains, which keeps the SMO protein inactive [86]. Upon HH stimulation or an active mutation (e.g., SMOM2), the π-cation lock is broken, allowing cholesterol; oxysterols, such as 20(S)-hydroxycholesterol [20(S)-OHC]; or cyclopamine to bind SMO CRD and cause CRD reorientation, further leading to the 7-transmembrane domain activation [86]. In support of this idea, the active form of SMO harbors a hydrophobic tunnel connecting the inner membrane leaflet with extracellular CRD domain [86], a potential pathway for cholesterol trafficking between CRD and 7-transmembrane domain. It waits for further investigation regarding how SMO CRD talks to the transmembrane domain to shuffle cholesterol between these domains.

It might be possible for other cholesterol derivatives or even other types of molecules to be involved in SMO activation. 24(S),25-epoxycholesterol (24(S),25-EC), a cilia oxysterol isolated form sea urchin embryo, interacts with and activates SMO [87]. Inhibition of the oxysterol biosynthesis enzyme, HSD11β2, by carbenoxolone (CNX, a derivative of the HSD11β2 inhibitor in licorice) or RNAi of the downstream enzyme Sterol 27-hydroxylase (CYP27A1) reduces HH signaling. Homozygous deletion of HSD11β2 reduces tumor weight of HH pathway-associated medulloblastoma [87]. Independent studies have also identified endogenous sterol-like densities associated with PTC1, including 24(S),25-epoxycholesterol (24(S),25-EC), 24-keto-cholesterol (24k-C), 25-hydroxycholesterol (25-OHC), and 24-hydroxycholesterol (24-OHC), which are characterized by mass spectrometry analysis [88,89]. Further cry-EM structure analysis indicates that 24(S),25-EC specifically interacts with a ligand binding site at seven-transmembrane helices of SMO during SMO–Gi coupling [90]. These studies suggest that 24(S),25-EC can function as trafficking molecular between PTC and SMO. Taken together, multiple molecules could be involved in SMO activation and subjected to PTC regulation. We expect that deeper mechanistic studies will address the regulation under physiological conditions in different types of tissues.

## 5. Perspectives

The question of how PTC inhibits SMO has been a long-standing puzzle. The involvement of small molecules, rather than a protein ligand, has been proposed [17] (Figure 2, left panel); however, the identity of these small molecules remain to be further characterized. The involvement of cholesterol may not fully answer the question of how PTC inhibits SMO because other molecules, such as phospholipids, are also involved. The biosynthesis of cholesterol by a cholesterol-producing enzyme DHCR7 near the ciliary base controls HH pathway activation [91]; however, it is unclear whether the endogenous cholesterol biosynthesis regulates HH singling in other tissues or other species, and whether HH signaling regulates the production of cholesterol. It is also unclear how PTC controls the pools of cholesterol so as to regulate SMO. Recent studies of the structural biology of SMO and PTC have made great contribution to the HH signaling field. Prediction of the structures of various SMO interacting proteins and molecules using the AlphaFold [92] may identify new mechanisms of SMO regulation. Although HH signaling depends on primary cilia in vertebrates but not in *Drosophila* [93], we have been convinced that the mechanisms of SMO regulation are largely conserved.

## Data Availability

Not applicable.
